# The relationship between teacher identity and learning engagement in sports training students: the mediating role of learning motivation

**DOI:** 10.3389/fpsyg.2026.1677374

**Published:** 2026-03-05

**Authors:** Hanzhen Ou, Ke Yang

**Affiliations:** 1 School of Physical Education Department, Central South University, Changsha, China; 2 School of Physical Education and Sports, Central China Normal University, Wuhan, China

**Keywords:** identity, learning engagement, learning motivation, physical education teacher, pre-service teachers

## Abstract

**Introduction:**

In the current educational context, the professional identity of physical education teachers serves as an important indicator of how prepared sports training students to enter the teaching profession.

**Methods:**

This study used structural equation modeling to analyze questionnaire data from 588 sports training students from nine universities in China. The aim was to examine the relationship between physical education teacher identity, motivation, and commitment to learning.

**Results:**

The overall level of physical education teacher identity among sports training students was moderately to high. A positive correlation was found between physical education teacher identity, learning motivation, and learning engagement. Furthermore, Physical education teacher identity was a significant predictor of sports training students' academic engagement, with learning motivation acting as a mediator in this relationship.

**Discussion:**

These findings offer valuable insights into how educational institutions can foster the development of physical education teacher identity by adapting their training programs, providing practical strategies for enhance students' motivation and engagement in learning.

## 1 Introduction

Students majoring in sports training represent an important reserve force for China's physical education. Although sports training programs are officially intended to prepare specialist coaches, evidence indicates that most graduates instead pursue careers as physical education teachers (Li et al., 2020). However, many of these students have relatively weak general academic foundations and have not received systematic, professional preparation in teacher education. Consequently, when they enter the school system, they may display noticeable deficiencies in pedagogical knowledge, instructional methods, and professional ethics.

Since the beginning of the 21st century, identity has increasingly been regarded as a core indicator of teacher professionalism and has been applied to studies of pre-service physical education teachers (Sachs, 2005; Zhou et al., 2016). The construction of professional identity among pre-service physical education teachers refers to guiding them, through appropriate pathways, to accurately understand the distinctive features of their vocation, develop sound professional awareness, and cultivate positive emotional engagement, thereby strengthening their commitment to a teaching career (Zhang et al., 2016). Research shows that a strong sense of teacher professional identity can enhance pre-service teachers' learning engagement (Peng and Zhang, 2024; Chen et al., 2020). Learning engagement is a positive, sustained affective state during learning, typically characterized by vigor, dedication, and absorption (Schaufeli et al., 2002). Students with higher learning engagement are more likely to adopt effective learning strategies and exhibit greater self-regulation (Luo et al., 2023). Numerous studies indicate that increased learning engagement not only improves students' educational experiences and academic performance, but also promotes their career development and critical thinking (Astin, 1993; Gurin et al., 2002; Fung et al., 2018). While theoretical frameworks posit a close relationship between pre-service teacher identity and learning engagement, students in sport-related majors experience distinct psychological and academic challenges compared to their peers in other disciplines (Nils et al., 2025). For example, NCAA athletes report that when academic demands encroach on training time, the perceived stagnation in the value of their physical capital, compounded by academic stress, can lead to burnout (Marangoni et al., 2023). This phenomenon is not limited to Western contexts. In China, students majoring in sports training often face similar psychological burnout due to unresolved conflicts between their primary identities as “student” and “athlete” (Yin et al., 2024). Concurrently, they must navigate the additional pressure of constructing a future professional identity, resulting in role transition stresses far exceeding those faced by traditional teacher education students.

A review of the existing literature reveals two primary gaps in this field. First, discussions on physical education teacher identity development have predominantly centered on pre-service teachers from standard education programs or in-service teachers, largely overlooking the significant cohort of students in sports training programs. Second, scholarly approaches have been primarily speculative or experience-based, lacking robust, large-scale quantitative analysis. Therefore, investigating the relationship between physical education teacher identity and learning engagement among sports training students holds dual significance. It not only complements and may refine existing general theories of teacher identity development but also informs practical improvements in curriculum reform and the development of a high-quality teaching workforce from non-traditional teacher training pathways.

## 2 Literature review

### 2.1 Physical education teacher identity and learning engagement

The pre-service stage is a critical period for forming a teacher identity. Serving as the psychological foundation for the transition from learner to teacher, it provides long-lasting motivation and influences teacher emotions (Timoštšuk and Ugaste, 2010). The identity construction of pre-service teachers involves continuous negotiation and integration between the student and teacher selves, and is characterized by its imaginative nature. This is reflected in their perceptions of the teaching role and professional practice, as well as the intensity of their desire to belong. Through pre-service learning, individuals progressively construct their professional identities, although these are often influenced by preconceived ideas (Britzman, 2003). Building upon Huang's (2021) framework of the cognitive, aspirational and volitional dimensions of pre-service teacher identity, this study defines pre-service physical education teacher identity as the psychological process by which individuals negotiate between their student and physical education teacher identities to form a coherent identity through cognitive, volitional and aspirational dimensions.

Identity theory posits that identity serves as the foundation for behavioral intentions and behavioral change, with an individual's identification with their self-concept shaping their behavioral choices. When learners possess clear career objectives, professional identity plays a significant role in their learning process (Klein et al., 2006). They are more inclined to engage in learning activities relevant to their future careers and are better able to approach the risks and adversities that may arise during the learning process with an optimistic attitude. Learning engagement not only serves as a crucial factor in motivating learners to develop learning motivation and achieve educational objectives (Fredricks et al., 2004), but also functions as a key indicator capable of reliably predicting learning achievement (Raza et al., 2020). Lu et al. (2025) confirmed that professional identity and learning engagement are positively correlated. Yang et al. (2025) found, through a survey of pre-service teachers in China, that professional identity significantly and positively predicted learning engagement: the stronger the pre-service teachers' sense of professional identity, the higher their level of learning engagement. Although previous research has not specifically examined the relationship between identity and learning engagement among pre-service physical education teachers, existing studies nevertheless provide valuable insights for this paper. Based on this, the first hypothesis proposed in this study is:

*H1*: Physical education teacher identity has a significant positive effect on the level of academic engagement of athletic training students.

### 2.2 The mediating role of learning motivation

Learning motivation constitutes a core theoretical foundation in educational psychology and learning sciences (Zhang and Hu, 2025). It refers to the composite of an individual's interests, expectations, goals, cognitive appraisals, and affective experiences related to learning activities (Pintrich, 2003). Learning motivation is typically categorized into intrinsic and extrinsic types. Intrinsic motivation arises from within the individual and is driven by inherent interest, curiosity, or the enjoyment of the learning process itself. In contrast, extrinsic motivation originates from external sources, such as the desire to obtain rewards, meet requirements, or avoid negative outcomes; it focuses more on the instrumental results of an action than on its inherent value (Deci and Ryan, 2008).

Learning motivation and career identity are well-established predictors of university students' academic performance (Jeno et al., 2018; Liu et al., 2012). Identity-based motivation theory offers a framework for understanding this link, positing that identity is crucial because it prepares individuals for meaning-making and guides their actions (Oyserman, 2007). Specifically, individuals are influenced by, and tend to act in accordance with the norms, expectations, and social narratives associated with their salient identities (Oyserman, 2009). Consequently, learning motivation can be understood as the key psychological mechanism through which a professional identity effectively sustains long-term learning behaviors (Matusovich et al., 2010). This view is supported empirically, with research identifying professional identity as a significant antecedent (Godwin and Kirn, 2020) and predictor (Liao et al., 2024) of student learning motivation. Rather than a simple construct, learning motivation is a complex system comprising dynamic factors such as desire, interest orientation, and volition (Sánchenz-Bolívar and Martínez-Martínez, 2022). For pre-service teachers, the aspiration to become a competent educator can serve as a foundational motivation, directing their energy toward learning activities aligned with the teaching profession (Zhang et al., 2016). Accordingly, a survey of pre-service teachers by Wei et al. (2013) revealed a significant positive correlation between teacher identity and learning motivation. Therefore, teacher identity may serve as a crucial motivational resource for students in sports training programs during their learning journey. Based on this, the study proposes a second hypothesis:

*H2*: Physical education teacher identity has a significant positive effect on motivation of athletic training students.

According to self-determination theory, motivation is a pivotal factor that initiates, sustains, and regulates individual behavior. It contributes positively by guiding students to fulfill academic responsibilities, achieve learning goals, and engage in deep learning (Rehman et al., 2020). Students with strong motivation tend to be more active in their studies—posing questions, seeking advice, mastering strategies, and participating in class—which is associated with lower levels of learning burnout (Alhadabi et al., 2019). Research further shows that students with strong intrinsic motivation achieve higher standardized test scores, demonstrate greater persistence, and produce higher quality work (Fredricks and McColskey, 2012; Lazowski and Hulleman, 2016). This is because intrinsic motivation arises from inherent interest in and satisfaction derived from the learning activity itself. When students find the process enjoyable or meaningful, they are more willing to invest time and effort, participate actively, complete assignments, and explore topics in depth (Flannery, 2017). Conversely, without motivational support, learning may devolve into a passive process characterized by aimless knowledge accumulation and rote memorization. Given that both learning motivation and engagement are central concerns in education, scholars widely recognize their close relationship (Yin and Wang, 2016) and affirm that motivation directly predicts and determines an individual's level of engagement (Walker et al., 2006). This link is corroborated by empirical evidence; for instance, a survey of Chinese university students confirmed a significant positive correlation between the two constructs (Xiang et al., 2022). Accordingly, highly motivated students typically exhibit greater enthusiasm for learning, stronger confidence in their academic abilities, and greater invested effort (Asikainen and Gijbels, 2017). Based on this theoretical and empirical foundation, the present study proposes the third and fourth hypotheses:

*H3*: There is a significant positive effect of motivation on athletic training students' engagement in learning

*H4*: The learning motivation of students majoring in sports training plays a mediating role between the identity of sports teachers and their learning engagement.

**Figure 1:**
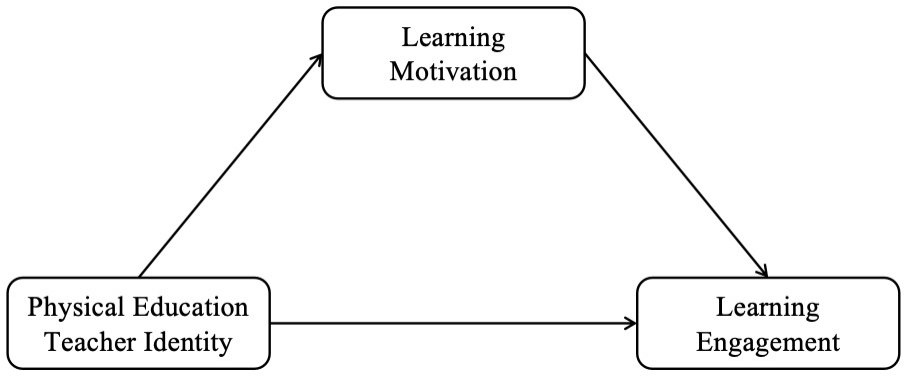
A theoretical model of the effect of physical education teacher identity on athletic sports students' academic engagement.

Figure 1 illustrates the theoretical model of the hypothesized relationship between physical education teachers' identity, motivation and commitment to learning.

## 3 Materials and methods

### 3.1 Data and participants

This study utilized a convenience sampling method to conduct a pilot survey, aiming to assess the quality of the questionnaire. Participants were students majoring in sports training from four universities in Hunan Province. While convenience sampling is widely adopted in academic research, it inherently limits the generalizability of findings, as the sample may not adequately represent broader social strata or geographical populations. Regarding sample size for pilot studies, it is recommended that the number of participants exceed 30 and be three to five times the number of items in the longest subscale of the questionnaire. Accordingly, 200 questionnaires were distributed for this pilot test. After applying standard data screening procedures, 153 valid responses were retained, resulting in a valid response rate of 76.5%.

To ensure the representativeness of the convenience sample, the subjects of the formal survey comprised students majoring in sports training at nine Chinese universities, including Wuhan Sports University, Xi'an Sports University, Central China Normal University, Fujian Normal University and Central South University. The selected institutions represent diverse types (comprehensive, teacher-training, and sports-specialized), all of which are well-ranked nationally, either institutionally or within their disciplines. This diversity was intended to secure a heterogeneous sample. Data collection was conducted online between September and October 2024. A total of 809 questionnaires were returned. Participants were assured of strict confidentiality, with all data anonymized and used for research purposes only. No personally identifiable information was disclosed. Following standard data screening procedures (e.g., removing incomplete responses and outliers), 588 questionnaires were deemed valid for final analysis. Thus, the effective response rate for analysis was 72.68%.

In terms of gender distribution, males accounted for 57.48% of the total student population, while females accounted for 42.52%. Since athletic training majors are a vital force in developing competitive sports talents in colleges and universities, this study included “whether they are currently training in sports teams” as one of the basic pieces of information. According to the data, 43.37% of the total number of students participated in training, while 56.63% did not. Considering that not all students majoring in athletic training have the intention to engage in the profession of physical education teacher, and that this study involves the investigation of the identity of physical education teachers, to ensure the rigor of the study, “whether they intend to engage in the profession of physical education teacher” was taken as a basic information, to carry out the subsequent analysis of differences. The data showed that 90.99% of the athletic training majors had the intention to teach, while only 9.01% of the students had no intention to teach. In terms of the distribution of institution types, 47.69% were from sports colleges and universities, 30.44% from teacher training colleges and universities, and 21.77% from comprehensive colleges and universities. In terms of grade distribution, first-year students accounted for 16.33%, sophomores for 25.68%, juniors for 25.17%, and seniors for 32.82%.

### 3.2 Measures

This questionnaire consists of two main parts. The first part covers basic information about students majoring in physical education, comprising a total of 5 questions, including students' gender, grade level, and type of institution. The second part consists of three scales: physical education teacher identity, learning motivation, and learning commitment.

#### 3.2.1 Physical education teacher identity scale

This study is based on domestic scholars' understanding of teachers' identity recognition. It draws on the open, mature scales developed by Wang et al. (2021), Wei (2008), Zhao et al. (2012), Zhou (2010), and Wang et al. (2010). It combines the characteristics of the sports training major student population to design the Physical Education Teacher Identity Recognition Scale. The scale consists of three dimensions—identity recognition, identity willingness, and identity expectations—with a total of 13 questions. Identity recognition includes 4 questions, such as “I can accurately explain to others what a physical education teacher is”; identity willingness consists of 5 questions, such as “I frequently participate in lectures, training, and other opportunities related to physical education teaching”; and identity expectations include 4 questions, such as “Physical education teachers should have a lifelong learning mindset.” The scale uses a five-point rating system ranging from “1-not at all” to “5-very much,” with higher scores indicating a higher level of physical education teacher identity among physical education majors. Initial research results showed a Cronbach's alpha of 0.927, a KMO value of 0.897, a significant Bartlett's sphericity test (*p* < 0.001), and an explained variance of 75.450%. The formal research results showed that Cronbach's alpha was 0.904, and the KMO measurement value was 0.916, indicating that the scale has good reliability and validity.

#### 3.2.2 Learning motivation scale

This study measures the learning motivation of students majoring in sports training using the internal and external motivation subscales from the Motivated Strategies for Learning Questionnaire (MSLQ) developed by Pintrich et al. (1993). This questionnaire is currently widely used in research on the learning motivation of Chinese university students. The questionnaire consists of two dimensions—internal motivation and external motivation—with a total of eight questions. Internal motivation includes four questions, such as “I enjoy the challenging content of the course, as it makes me feel like I am learning new knowledge”; external motivation includes four questions, such as “Getting a good grade in this course is what satisfies me the most right now.” The scale uses a seven-point rating system ranging from “1-Strongly Disagree” to “7-Strongly Agree,” with higher scores indicating higher levels of learning motivation among physical education students. Preliminary research results showed a Cronbach's alpha of 0.887, a KMO value of 0.881, and a significant Bartlett's sphericity test (*p* < 0.001), with an explained variance of 79.840%. Formal research results showed a Cronbach's alpha of 0.850 and a KMO measurement value of 0.860, indicating that the scale has good reliability and validity.

#### 3.2.3 Learning investment scale

This study measured learning engagement using the Chinese version of the Utrecht Work Engagement Scale for Students (UWES-S), adapted by Fang et al.'s (2008). This scale has demonstrated good reliability, validity, and cultural appropriateness for Chinese university students in prior research. It consists of 17 items across three subscales: vigor (6 items, e.g., “I feel full of energy when I am studying”), dedication (5 items, e.g., “I am enthusiastic about my studies”), and absorption (6 items, e.g., “Time flies when I am studying”). All items were rated on a 7-point Likert scale from 1 (Strongly Disagree) to 7 (Strongly Agree), with higher scores indicating greater learning engagement. Data from the pilot study (*n* = 153) were first analyzed to examine the scale's psychometric properties. The scale showed excellent internal consistency (Cronbach's α = 0.974). The Kaiser–Meyer–Olkin (KMO) measure was 0.946, and Bartlett's test of sphericity was significant (*p* < 0.001), confirming the data's suitability for factor analysis. An exploratory factor analysis using orthogonal rotation was conducted. Items were retained based on the following criteria: (a) a primary factor loading >0.50, and (b) no significant cross-loading (defined as a loading >0.50 on another factor with a difference of < 0.20 from the primary loading). The item “I find it hard to detach myself from my studies” was removed due to cross-loading issues. The remaining 16 items loaded cleanly onto three distinct factors, which together accounted for 85.43% of the total variance. A subsequent confirmatory factor analysis supported the good fit of this three-factor structure, evidencing its construct validity. Thus, the adapted scale demonstrated high reliability and validity in the pilot phase. In the main study (*N* = 588), the refined 16-item scale maintained strong psychometric properties, with a Cronbach's α of 0.923 and a KMO value of 0.943, confirming its robustness for use with the target population.

#### 3.2.4 Data analysis

This study utilized SPSS 29.0 to assess the reliability and validity of the three scales, and performed descriptive statistics and correlation analysis on the data. Mediation analysis was conducted using the online data analysis platform SPSSAU.

## 4 Results

### 4.1 Common method bias test

This study assessed common method bias using Harman's single-factor test. An unrotated exploratory factor analysis of all measurement items yielded eight factors with eigenvalues greater than 1, with the first factor accounting for only 32.955% of the total variance, which is below the recommended threshold of 40%. Furthermore, a confirmatory factor analysis specifying a single-factor model showed a poor fit to the data (χ^2^/*df* = 1.200, RMSEA = 0.018, CFI = 0.990, TLI = 0.988, NFI = 0.941). Based on these two analytical approaches, we concluded that common method bias was not a severe concern in this study and would not substantially affect the subsequent data analyses.

### 4.2 Descriptive statistics and correlation analyses

Table 1 lists the descriptive statistics and variable correlations between the study variables. The results indicate a significant positive correlation between physical education teacher identity, learning motivation, and learning engagement among students majoring in physical education training.

**Table 1:** Variable correlations and descriptive statistics for study variables.

**Variables**	***M***	***SD***	**1**	**2**	**3**	**4**	**5**	**6**	**7**	**8**	**9**	**10**	**11**
Identity	3.70	0.82	1										
Identity awareness	3.67	0.97	0.809^**^	1									
Identity expectations	3.71	1.01	0.855^**^	0.549^**^	1								
Identity intentions	3.70	1.03	0.784^**^	0.482^**^	0.473^**^	1							
Learning motivation	4.24	1.16	0.506^**^	0.446^**^	0.409^**^	0.392^**^	1						
Internal motivation	4.34	1.38	0.427^**^	0.358^**^	0.347^**^	0.346^**^	0.844^**^	1					
External motivation	4.15	1.37	0.426^**^	0.394^**^	0.342^**^	0.314^**^	0.842^**^	0.421^**^	1				
Learning engagement	4.34	1.16	0.565^**^	0.447^**^	0.488^**^	0.446^**^	0.492^**^	0.440^**^	0.389^**^	1			
Vitality	4.48	1.29	0.453^**^	0.370^**^	0.406^**^	0.328^**^	0.421^**^	0.388^**^	0.322^**^	0.843^**^	1		
Dedication	4.18	1.46	0.491^**^	0.375^**^	0.413^**^	0.413^**^	0.388^**^	0.375^**^	0.279^**^	0.852^**^	0.584^**^	1	
Attention	4.35	1.40	0.475^**^	0.377^**^	0.404^**^	0.381^**^	0.425^**^	0.341^**^	0.377^**^	0.813^**^	0.507^**^	0.557^**^	1

^*^p < 0.05, ^**^p < 0.01.

As shown in Table 1, students majoring in physical education have relatively high levels of learning motivation (*M* = 4.24) and learning engagement (*M* = 4.34), while their sense of identity as physical education teachers is relatively low (*M* = 3.70). Among these, the differences between identity recognition (*M* = 3.67), identity expectations (*M* = 3.71), and identity (*M* = 3.70) are relatively small. Additionally, the correlations between physical education teacher identity and learning motivation and learning engagement are 0.506 and 0.565, respectively, both exceeding 0.5, indicating a significant correlation among the variables.

### 4.3 Analysis of the mediating role of learning motivation

**Figure 2:**
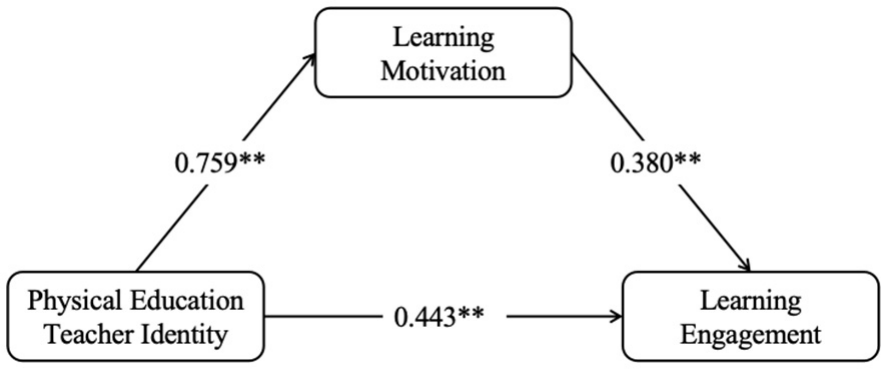
Path diagram of the mediation mode. *p < 0.001, **p < 0.01.

This study utilized SPSS AU as a tool and applied structural equation modeling for parameter estimation to explore the mediating effect of learning motivation. Physical education teacher identity was set as the predictor variable, learning motivation as the mediating variable, and learning engagement as the outcome variable. The model demonstrated good fit after controlling for latent variables including gender, institution type, and grade level (χ^2^/*df* = 1.628, GFI = 0.984, CFI = 0.985, TLI = 0.969, IFI = 0.985, RMSEA = 0.033, SRMR = 0.025). The standardized path analysis results are shown in Figure 2. Additionally, bootstrap methods with robust standard errors were applied to test the significance of mediating effects. A bias-corrected bootstrap test (5,000 iterations) was performed to check whether the indirect paths shown in Figure 1 were significant. In the test, the 95% confidence interval of the indirect path coefficient did not include 0, indicating statistical significance. Using CFA to measure the three latent variables of identity, learning motivation and learning engagement, the results showed that the CR values for the three latent variables were 0.904, 0.851 and 0.923 respectively, with AVE values of 0.608, 0.506 and 0.656 respectively.

Three direct effect pathways exist among the three latent variables in this study: physical education teacher identity → learning motivation, learning motivation → learning engagement, and physical education teacher identity → learning engagement. As shown in Table 2, the standardized coefficients for each path were 0.715, 0.276, and 0.598 respectively, with all three paths being statistically significant at the *p* < 0.001 level. Consequently, Hypotheses H1, H2, and H3 were all supported.

**Table 2:** Path testing of direct effects of each variable.

**Path**	**Unstandardized path coefficient**	**Standardized path coefficient**	***SE***	***CR***	***p***
Identity → learning motivation	0.715	0.506	0.050	14.22	< 0.001
Learning motivation → learning engagement	0.276	0.277	0.038	7.332	< 0.001
Identity → learning engagement	0.598	0.425	0.053	11.242	< 0.001

To ensure the validity of the results, bootstrapping was employed for verification in this study. The test results are presented in Table 3. Regarding indirect effects, the indirect effect of physical education teacher identity on learning commitment via learning motivation was 0.197, with a 95% confidence interval not containing zero (0.135, 0.265) and an effect size of 0.598. Consequently, Hypothesis H4 holds: learning motivation mediates the relationship between physical education teacher identity and learning commitment.

**Table 3:** Testing the mediating effect of learning motivation.

**Path**	**Effect**	**95% CI**		***SE***
		**Lower limit**	**Upper limit**	
Identity → learning motivation → learning engagement (indirect effects)	0.197	0.135	0.265	0.033
Identity → learning engagement (direct effects)	0.598	0.493	0.702	0.053
Identity → learning engagement (total effect)	0.795	0.701	0.889	0.048

## 5 Discussion

This study first measured the levels of physical education teacher identity, learning motivation, and learning engagement among students majoring in physical education. Subsequently, the relationships among these three factors were examined, and the mediating role of learning motivation was further verified.

### 5.1 Level of identity recognition among physical education teachers

Identity theory suggests that individuals derive personal purpose and meaning by understanding how they can contribute to society (Caza and Creary, 2016). However, for students majoring in sports training, the mean scores across all dimensions of physical education teacher identity fall below 3.75. Although this indicates an above-average level, it highlights a need for significant improvement. This relatively moderate identification may stem from several factors specific to their dual role. First, as student-athletes training to become physical education teachers, their cognitive, emotional, and behavioral engagement with teaching often differs from that of in-service teachers. This discrepancy can create conflict during their professional identity formation. Within the school environment, their primary focus and source of identity is often that of a “competent athlete,” a role which can marginalize other professional identities (Maivorsdotter et al., 2014). Second, the explicit goal of most sports training programs is to cultivate coaches, not teachers. Consequently, the curriculum, teaching materials, and pedagogical philosophy are predominantly oriented toward coaching, providing few opportunities or incentives to develop a strong identity as a physical education teacher. Finally, the distinct societal expectations for coaches and teachers—affecting perceived duties and work styles—can lead to internalized role conflict (O'Connor and MacDonald, 2002), further complicating the identity construction process for these students.

### 5.2 The impact of physical education teachers' identity recognition on learning engagement

Teacher professional identity functions as a regulatory mechanism influencing student learning behaviors. According to social identity theory, identity provides the psychological drive for the initiation and maintenance of behavior. In the professional domain, occupational identity—a manifestation of social identity—plays a crucial role in shaping, sustaining, and guiding an individual's professional conduct (Jeanson and Michinov, 2020). Consistent with this theory, the findings of this study indicate a positive correlation between the level of physical education teacher identity and the level of learning engagement among sports training majors. As prior research suggests, achieving a sense of teacher identity is associated with more proactive learning attitudes and greater engagement (Mei and Wei, 2022; Zhang et al., 2016). Reinforcement theory offers further support, positing that sustained stimuli can alter cognitive and behavioral responses, thereby increasing the probability of specific behaviors (Liu et al., 2023). However, the current study also reveals a notable issue: the learning engagement level of these students significantly surpasses their self-reported physical education teacher identity level. Previous studies suggest that pre-service teachers' substantial investment in learning can, in turn, enhance their sense of professional identity (Ye et al., 2021). Therefore, the observed discrepancy in the present sample may still be largely attributable to the primary program goal of cultivating coaches, which results in a relative lack of targeted teacher education. This suggests that while a strong sense of physical education teacher identity provides the essential cognitive and motivational foundation for proactive learning among sports training majors, the content of their learning must also be explicitly connected to teacher professional development. Such alignment is necessary to foster a mutually reinforcing relationship between professional identity and learning engagement.

### 5.3 The mediating role of learning motivation

This study found that learning motivation partially mediates the relationship between physical education teachers' professional identity and students' learning engagement among sports training. For instance, research on teacher education students indicates that a stronger professional identity is associated with greater learning motivation, which in turn fosters increased enthusiasm, concentration, and diligence in mastering knowledge and skills (Lin, 2019; Zhang et al., 2011). Learning motivation provides the directed impetus for action, while learning engagement represents the goal-oriented process through which this impetus is manifested. Accordingly, students with strong motivation typically exhibit greater enthusiasm for learning, stronger confidence in their academic abilities, and a greater investment of effort (Asikainen and Gijbels, 2017). Furthermore, extensive research confirms that purposeful learning activities positively influence university students' learning outcomes (Xu et al., 2020). Therefore, the level of physical education teacher identity among sports training majors not only directly affects their learning engagement but also exerts an indirect effect through the mediating pathway of learning motivation.

### 5.4 Research implications

Grounded in the reality of expanded enrolment in Chinese sports training programs and the trend of graduates increasingly pursuing careers as physical education teachers, this study investigated this specific cohort across three key dimensions: professional identity, learning motivation, and learning engagement. The findings provide an empirical basis for curriculum reform and address a notable gap in the existing literature. The implications are threefold. First, while a significant number of students express a willingness to become PE teachers, the current singular focus on coaching within their training creates a mismatch. Therefore, targeted policy interventions and enhanced teacher education components are needed within sports training programs to ensure the future quality of China's physical education teaching workforce. Second, a strong sense of PE teacher identity is positively correlated with students' learning engagement. Consequently, training institutions should aim not only to develop students' knowledge structures but also to foster their psychological and professional identity development. Third, moving beyond prior studies that examined PE teacher identity in isolation, this research integrates the critical factor of student-athletes' learning engagement. This approach helps ground the discussion of identity development in tangible learning behaviors, thereby avoiding theoretical abstraction.

### 5.5 Limitations and future research

While this study offers valuable insights into the development of students majoring in sports training through empirical investigation, it is not without limitations. First, the reliance on quantitative data means that in-depth interviews with students were not conducted. Consequently, the study lacks the rich qualitative insights needed to more deeply explore and interpret the distinctive characteristics of this group. Future research should employ qualitative methods, such as grounded theory, to enhance understanding and provide a deeper analysis. This would allow for further testing, revision, and development of the theoretical model proposed here, thereby strengthening its applicability and scientific robustness. Second, the current model primarily examines the role of learning motivation, without delving into other potential psychological and contextual factors that influence learning behaviors. Subsequent studies should investigate the interactions among a broader set of variables to further enrich and refine the model. This expanded scope is necessary to more comprehensively reveal the underlying mechanisms at play.

## 6 Conclusion

The results indicate that physical education teachers' recognition of their identity is generally at a moderately high level. There is a positive correlation between physical education teachers' identity recognition, learning motivation, and learning engagement. Physical education teachers' identity recognition has a significant predictive effect on the learning engagement of students majoring in sports training, with learning motivation serving as a mediating factor in this relationship.

## Data Availability

The original contributions presented in the study are included in the article/Supplementary material, further inquiries can be directed to the corresponding author.
