# Supplementary material for: The relationship between teacher identity and learning engagement in sports training students: the mediating role of learning motivation
Source: Front Psychol. 2026 Mar 5;17:1677374. doi: 10.3389/fpsyg.2026.1677374 (PMC12999411; doi:10.3389/fpsyg.2026.1677374)
Supplement: Supplementary file 1 [file Data_Sheet_1.docx]

**Supplementary Material A**

| **Dimension** | **Title** |
| --- | --- |
| Identity Perception | I can accurately explain the role of a physical education teacher to others (Wang Ye'an, 2021). |
|  | The professional value of a physical education teacher aligns with their personal value (Wang Ye'an, 2021). |
|  | I believe the work of physical education teachers is important for promoting students' growth and development (Wei Shuhua, 2008). |
|  | I consistently compare my personal identity with the identity of a teacher from a teacher's perspective (self-developed). |
| Identity Commitment | I often pay attention to the teaching methods used by instructors in class (Zhao Hongyu, 2012). |
|  | I frequently follow information related to physical education teachers, such as policies, benefits, healthcare, and professional development opportunities (Zhao Hongyu, 2012). |
|  | I regularly participate in lectures, training sessions, and other opportunities related to physical education teaching (Zhao Hongyu, 2012). |
|  | I am willing to undertake tasks that benefit students, even if they may not be compensated (Zhou Ke, 2010). |
|  | If given the opportunity to choose another profession after graduation, I would still choose to become a physical education teacher (Wang Xinqiang, 2012). |
| Identity Expectation | I aspire to become a popular teacher (Zhou Ke, 2010). |
|  | I believe I can become an excellent physical education teacher (Wang Xinqiang, 2012). |
|  | I aim to cultivate students' interest in physical education (Zhou Ke, 2010). |
|  | Physical education teachers should embrace the concept of lifelong learning (Zhou Ke, 2010). |
